# Thermosensitive TRPV4 channels mediate temperature-dependent microglia movement

**DOI:** 10.1073/pnas.2012894118

**Published:** 2021-04-22

**Authors:** Rei Nishimoto, Sandra Derouiche, Kei Eto, Aykut Deveci, Makiko Kashio, Yoshitaka Kimori, Yoshikazu Matsuoka, Hiroshi Morimatsu, Junichi Nabekura, Makoto Tominaga

**Affiliations:** ^a^Division of Cell Signaling, National Institute for Physiological Sciences, National Institutes of Natural Sciences, 444-8787 Okazaki, Japan;; ^b^Department of Physiological Sciences, The Graduate University for Advanced Studies (SOKENDAI), 444-8585 Okazaki, Japan;; ^c^Department of Anesthesiology and Resuscitology, Okayama University Hospital, 700-8558 Okayama, Japan;; ^d^Thermal Biology Group, Exploratory Research Center on Life and Living Systems, National Institutes of Natural Sciences, 444-8787 Okazaki, Japan;; ^e^Division of Homeostatic Development, National Institute for Physiological Sciences, National Institutes of Natural Sciences, 444-8585 Okazaki, Japan;; ^f^Department of Physiology, Aichi Medical University, 480-1195 Nagakute, Japan;; ^g^Department of Management and Information Sciences, Faculty of Environmental and Information Sciences, Fukui University of Technology, 910-8505 Fukui, Japan;; ^h^Department of Anesthesiology and Resuscitology, Okayama University Graduate School of Medicine, Dentistry, and Pharmaceutical Sciences, 700-8558 Okayama, Japan;; ^i^Institute for Environmental and Gender-Specific Medicine, Juntendo University, 279-0021 Chiba, Japan

**Keywords:** microglia, movement, TRP channels, TRPV4

## Abstract

Microglia maintain central nervous system homeostasis by monitoring changes in their environment (resting state) and by taking protective actions to equilibrate such changes (activated state). These surveillance and protective roles both require constant movement of microglia. Interestingly, induced hypothermia can reduce microglia migration caused by ischemia, suggesting that microglia movement can be modulated by temperature. Although several ion channels and transporters are known to support microglia movement, the precise molecular mechanism that regulates temperature-dependent movement of microglia remains unclear. Some members of the transient receptor potential (TRP) channel superfamily exhibit thermosensitivity and thus are strong candidates for mediation of this phenomenon. Here, we demonstrate that mouse microglia exhibit temperature-dependent movement in vitro and in vivo that is mediated by TRPV4 channels within the physiological range of body temperature. Our findings may provide a basis for future research into the potential clinical application of temperature regulation to preserve cell function via manipulation of ion channel activity.

## Significance

Movement is a key feature of the surveillance and protective roles of microglia. This dynamic process is highly modulated by the surrounding environment. We discovered that microglia movement is temperature dependent in vitro and in vivo. Our investigation of thermosensitive TRP channel involvement in this phenomenon revealed several candidates including TRPM2, TRPM4, and TRPV4 channels. Using pharmacological tools and transgenic mice, we showed that the temperature dependency of microglia movement mainly relies on TRPV4 channel activity. Understanding the mechanisms by which temperature modulates microglia movement will improve our comprehension of pathological processes and allow the identification of new leads for the treatment of brain pathologies.

Microglia constitute resident macrophages in the central nervous system (CNS). They play a key role in CNS homeostasis by monitoring changes in their environment (resting state) and by taking protective actions to equilibrate these changes (activated state) (1, 2). These surveillance and protective roles both require constant movement of microglia, either through protrusion of processes or migration to affected areas (1, 3). Interestingly, inducing hypothermia reduces the movement of activated microglia (4, 5), suggesting that microglia can sense temperature and might be modulated by temperature changes.

Thermosensation is an essential property for the survival of living organisms and is critical even at the cellular level (6). Indeed, temperature changes in the microenvironment surrounding cells can constitute a signal that modulates cellular behavior (7–9). As an activity that integrates functions of the cell membrane, membrane proteins, cytoskeleton, cytosol, and extracellular matrix, cell motility is particularly sensitive to such perturbations (10, 11). As early as 1971, increases in temperature were shown to be directly responsible for increased motility of leukocytes (12). However, the molecular mechanism underlying this temperature-dependent modulation of cell motility remains elusive.

Transient receptor potential (TRP) channels are largely nonselective cation channels that sense chemical and mechanical stimuli as well as temperature (13–15). The TRP channel superfamily is subdivided into seven subfamilies: TRPV (vanilloid), TRPC (canonical), TRPM (melastatin), TRPML (mucolipin), TRPN (NOMPC), TRPP (polycystin), and TRPA (ankyrin) based on their primary amino acid sequences (16, 17). Among the 28 different mammalian TRP channels, 11 members exhibit temperature sensitivity, and each has a different temperature threshold for activation within the range of physiological temperatures (6). Most TRP channels, including thermosensitive TRP channels, have a high permeability to calcium and sodium (18). Ion fluxes are known to drive and modulate different types of cell movements such as migratory responses (19). Therefore, cation fluxes mediated by thermosensitive TRP channels could form the basis for temperature-mediated cell movement.

Since microglia exhibit an inherent ability to migrate upon exposure to adverse environments, in the present study, we investigated whether temperature can modulate microglia movement in vitro and in vivo. We also sought to determine the contribution of thermosensitive TRP channels to microglial thermosensation. We demonstrate that mouse microglia exhibit temperature-dependent changes in movement that are likely mediated by activity of thermosensitive TRPV4 within the physiological range of body temperature. Our findings provide a basis for future research into the potential clinical application of temperature regulation to preserve cell function.

## Results

### Microglia Exhibit Temperature-Dependent Motility In Vitro.

**Fig. 1.**
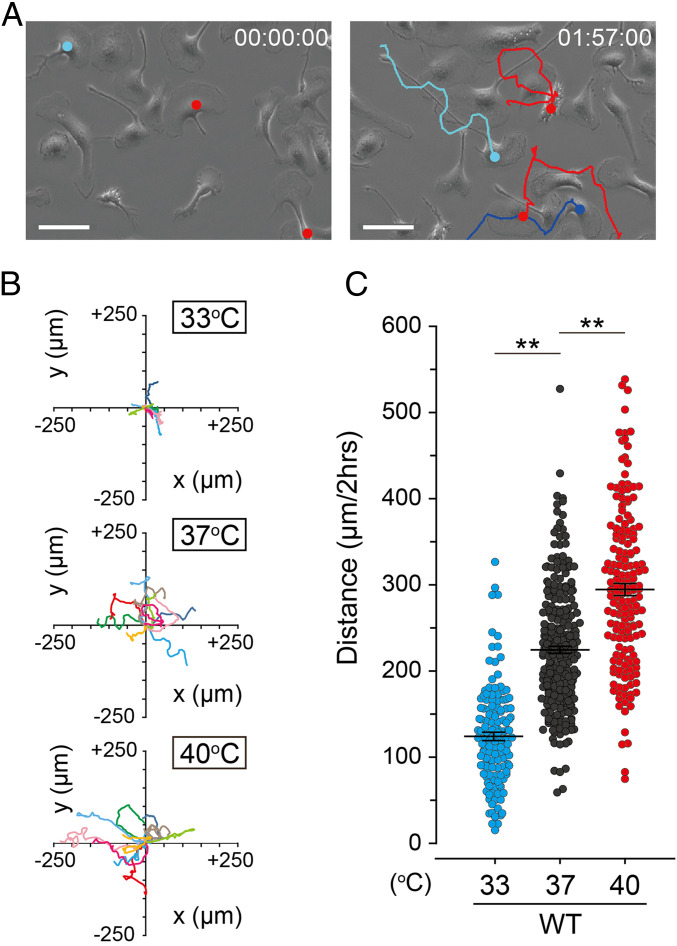
Microglia exhibit temperature-dependent motility in vitro. (*A*) Representative images at 37 °C from time-lapse imaging (Movie S1). Images taken at time 0 and 1 h 57 min are shown on the left and right, respectively. Filled colored circles indicate *x*,*y* coordinates of a representative target and colored lines indicate trajectory. The trajectories were superimposed on the images using the ImageJ Manual tracking plugin. (Scale bar represents 50 μm.) (*B*) Trajectories of 10 representative microglia over 2 h at 33 °C, 37 °C, and 40 °C. Paths are arranged to show origins at *x* = *y* = 0. Each line indicates the trajectory of one cell. (*C*) Temperature-dependent changes in distance traveled by primary mouse microglia at 33 °C (*n* = 137), 37 °C (*n* = 181), and 40 °C (*n* = 171). Filled circles indicate migration distance of each microglia over 2 h. Horizontal lines indicate mean ± SEM **P < 0.01 (one-way ANOVA followed by post hoc Bonferroni tests for multiple comparisons).

We first investigated whether microglia can detect and respond to changes in temperature in vitro using wild-type (WT) microglia primary cultures derived from mouse pups. We established a temperature-controlled time-lapse imaging system that allows long-term observation of microglia movements at a single-cell level in response to consecutive temperature changes (Movie S1). Within minutes of shifting the temperature from 37 °C to 33 °C and 40 °C, microglia movement reversibly slowed and accelerated, respectively. Typical microglia exhibit random motility and have a fan-shaped cell body with an elastic tail that appears to be polarized at 37 °C (Fig. 1*A*). Analysis of the trajectories of 10 typical cells over 2 h at 33 °C, 37 °C, or 40 °C showed that microglia moved more with increasing temperature (Fig. 1*B*). To exclude possible adverse cellular effects associated with an extended incubation period, we performed time-lapse imaging of mouse microglia over 2 h at three different temperature conditions using fixed time schedules. Again, the distance traveled by microglia showed significant temperature-dependent differences (124.2 ± 4.9 μm at 33 °C, 224.6 ± 4.1 μm at 37 °C, and 294.6 ± 7.0 μm at 40 °C; Fig. 1*C*). This result indicated that mouse microglia have a regulatory mechanism that allows detection of changes in the environmental temperature that translate to altered motility.

### TRPM2, TRPM4, and TRPV4 Channels Are Functionally Expressed in Microglia.

**Fig. 2.**
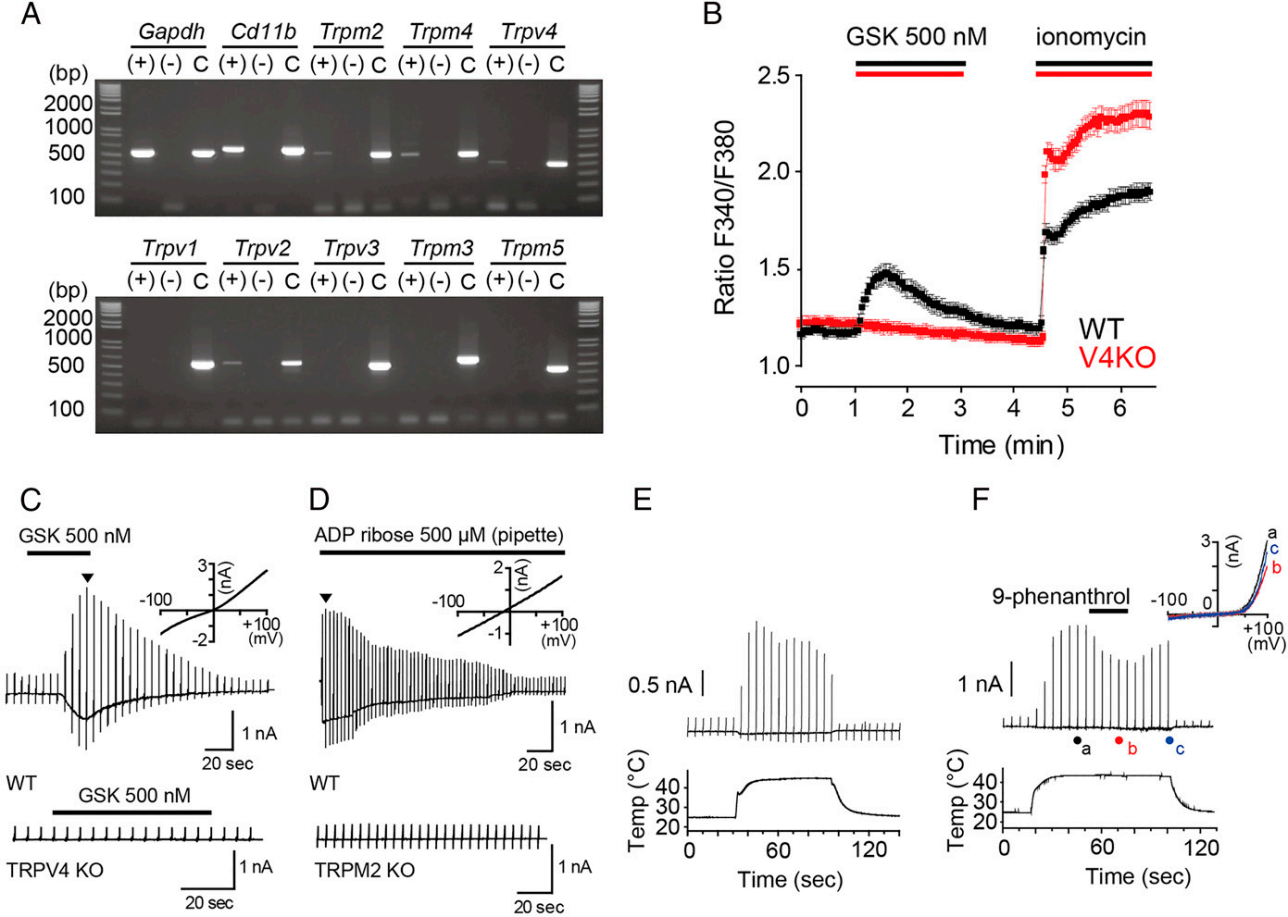
TRPM2, TRPM4, and TRPV4 channels are functionally expressed in microglia. (*A*) Expression patterns of eight thermosensitive TRP channel genes in WT microglia and two controls (Gapdh and Cd11b, a microglia marker). *C* indicates amplified fragments using each cDNA as a template. (*B*) Mean calcium imaging traces in primary microglia from WT (black, *n* = 36) and TRPV4KO (red, *n* = 27) mice. Data are represented as mean ± SEM. GSK indicates treatment with the TRPV4 activator GSK-1016790A (500 nM). Ionomycin (5 μM) was applied to assess cell viability. (*C*) Representative whole-cell current traces of GSK-1016790A (GSK, 500 nM)–evoked responses in WT microglia (*Top*, *n* = 7 cells) and TRPV4KO microglia (*Bottom*, *n* = 6 cells) with ramp pulses from −100 mV to 100 mV every 5 s; Vm = −60 mV. *Inset* shows IV relationship at the arrowhead. (*D*) Representative whole-cell current traces of ADP ribose (500 μM)–evoked responses in WT microglia (*Top*, and *n* = 3 cells) and TRPM2 KO microglia (*Bottom*, *n* = 3 cells) with ramp pulses from −100 mV to 100 mV delivered every 3 s; Vm = −60 mV. *Inset* shows IV relationship at the arrowhead. (*E* and *F*) Representative whole-cell traces of heat-evoked currents in WT microglia in the absence (*E*) or presence (*F*) of 9-phenanthrol (*Upper*, 100 μM) with ramp pulses from −100 mV to 100 mV delivered every 5 s. Lower trace indicates temperature transition. Vm = −70 mV. *Inset* shows IV relationships at the corresponding letters.

To find candidate genes that are involved in temperature-dependent microglia movement, we next focused on the TRP channel superfamily (20). Several TRP channels exhibit thermosensitivity, and the temperature thresholds vary by channel type (18). Trpm2, Trpm4, Trpv4, and Trpv2 messenger ribonucleic acid expression was detected in primary microglia (Fig. 2*A*). For Trpm2 and Trpv4, the RT-PCR and protein expression patterns we observed were consistent with previous results (*SI Appendix*, Fig. S1 *A*–*C*) (21, 22). Although TRPV2 was previously reported to be functionally expressed in microglia (23), its temperature threshold for activation exceeds the physiological range (>52 °C) (24). Thus, TRPV2 is likely not involved in temperature-dependent microglia movement, and we did not examine this channel further. We next used Ca^2+^-imaging and whole-cell patch-clamp methods to examine whether the other three TRP channels function in mouse microglia. In Ca^2+^-imaging, we observed intracellular Ca^2+^ increases in WT microglia following application of the selective TRPV4 activator GSK-1016790A (GSK, 500 nM) in an extracellular Ca^2+^-dependent manner (*SI Appendix*, Fig. S1*D*) (Fig. 2*B*), indicating that the intracellular Ca^2+^ increases are due to Ca^2+^ influx probably through TRPV4, while no such responses occurred in Trpv4-knockout (V4KO) microglia. Whole-cell patch-clamp analysis revealed that GSK application resulted in a robust current in WT, but not in V4KO, primary mouse microglia (Fig. 2*C*), indicating TRPV4 activation and supporting the Ca^2+^-imaging results. Meanwhile, in WT but not Trpm2-knockout (M2KO), primary mouse microglia in a whole-cell configuration, intracellular application of adenosine diphosphate ribose (ADP ribose, 500 μM), a TRPM2 activator, induced large currents that were indicative of TRPM2 activation (Fig. 2*D*). For the Ca^2+^-impermeable TRPM4 channel, heat and intracellular Ca^2+^ are natural stimuli (25). Since specific activators are not available for this channel, we evaluated whether 9-phenanthrol, a supposedly specific TRPM4 inhibitor (26), could inhibit heat-evoked currents observed in primary microglia in patch-clamp recordings. Upon heat stimulus, robust, outwardly-rectifying currents were observed in primary microglia (Fig. 2*E*). However, in the presence of intracellular Ca^2+^ in a concentration that was sufficient to activate TRPM4, heat-evoked currents in primary microglia were partially inhibited by application of 9-phenanthrol (100 μM), indicating that TRPM4 could be functionally expressed (Fig. 2*F*) (*SI Appendix*, Fig. S2). Together, these results suggested that TRPM2 and TRPV4 could function in mouse microglia. The role of TRPM4 in microglia movement is still unclear, even though microglia did exhibit heat sensitivity in vitro.

### Genetic Elimination of Trpm2 or Trpm4 Results in Lower Motility of Microglia Compared with WT.

**Fig. 3.**
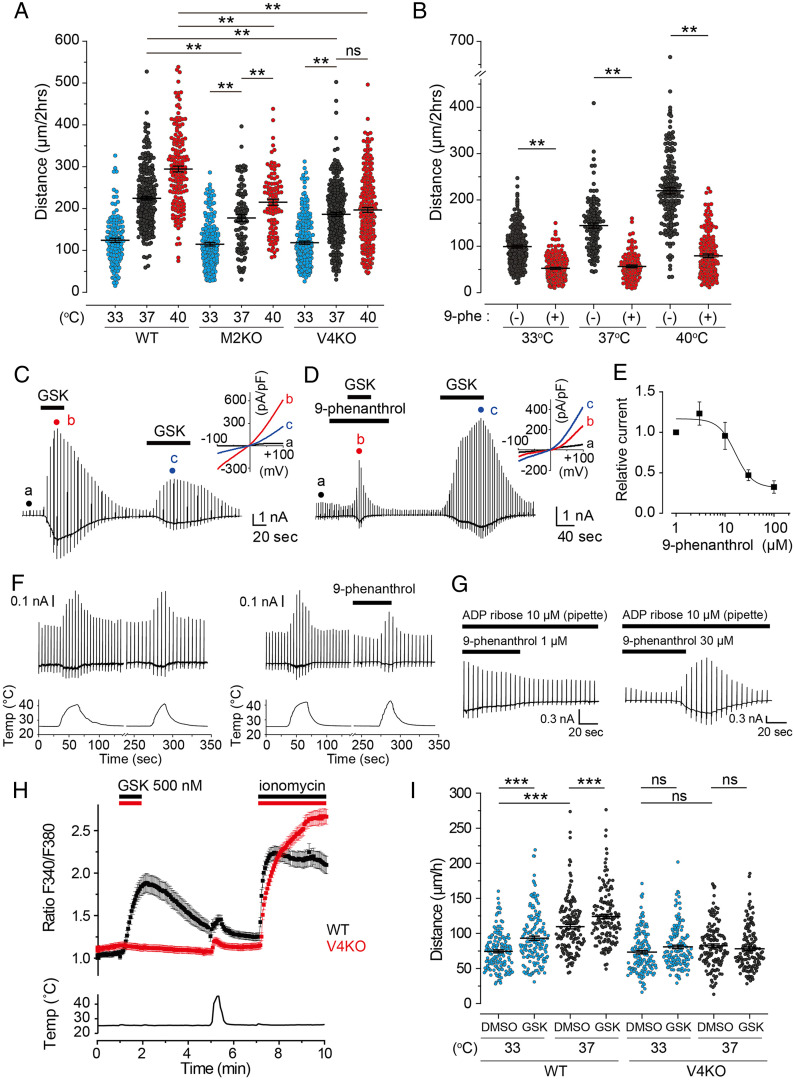
Genetic elimination of Trpm2 or Trpm4 and 9-phenanthrol, a TRPM4 inhibitor, affects temperature-dependent microglia movement. (*A*) Average distances of migrating microglia isolated from WT, M2KO, or V4KO mice exposed to 33 °C (M2KO, *n* = 128; V4KO, *n* = 236), 37 °C (M2KO, *n* = 99; V4KO, *n* = 262), or 40 °C (M2KO, *n* = 102; V4KO, *n* = 217). WT data are shown in Fig. 1*C*. Filled circles indicate migration distance for each microglia cell over 2 h. Horizontal lines indicate mean ± SEM. At 33 °C, 37 °C, and 40 °C, M2KO microglia moved 115.0 ± 4.2 μm, 177.7 ± 7.9 μm, and 215.4 ± 7.2 μm, respectively, whereas V4KO microglia moved 118.6 ± 3.8 μm, 186.4 ± 4.6 μm, and 196.8 ± 5.9 μm at those respective temperatures. **P < 0.01 (two-way ANOVA followed by post hoc Bonferroni tests for multiple comparisons). (*B*) Inhibition of temperature-dependent microglia movement by 9-phenanthrol (30 μM) at 33 °C (*n* = 212), 37 °C (*n* = 124), and 40 °C (*n* = 187). (−) indicates the control distance before application of 9-phenanthrol at each temperature condition. Filled circles indicate migration distance for each microglia cell over 2 h. Horizontal lines indicate mean ± SEM. Mean values are 99.5 ± 2.8 μm, 144.7 ± 5.1 μm, and 220.0 ± 6.0 μm versus 52.5 ± 1.7 μm, 56.6 ± 2.5 μm, and 79.6 ± 3.4 μm in the presence and absence of 9-phenanthrol at 33 °C, 37 °C, and 40 °C, respectively. **P < 0.01 (two-way ANOVA followed by post hoc Bonferroni tests for multiple comparisons). (*C* and *D*) Representative whole-cell trace of GSK (100 nM)-induced currents in absence and presence of 9-phenanthrol (30 μM) inhibitor upon ramp pulses from −100 mV to 100 mV in a HEK293T cell expressing mouse TRPV4; (*Inset*) Ramp-pulse responses at *A*, *B*, and *C* are shown as an IV relationship. Vm = −60 mV. (*E*) Concentration-dependent inhibition of TRPV4 currents by 9-phenanthrol. The maximum GSK (100 nM)-induced inward current in the presence of 9-phenanthrol was normalized to currents observed in the presence of 1 μM 9-phenanthrol. The results were fitted with a logistic curve (*n* = 6 to 8). Data are represented as mean ± SEM. (*F*) Representative whole-cell traces of heat-evoked currents in a HEK293T cell expressing mouse TRPV4 without (left) and with 9-phenanthrol (right, 30 μM) treatment. *n* = 3 for each. Vm = −60 mV. (*G*) Dose-dependent inhibition of ADP ribose (10 μM)–induced current by 1 μM (left) and 30 µM (right) 9-phenanthrol in a HEK293T cell expressing mouse TRPM2. Vm = −60 mV. (*H*) Calcium imaging traces in primary microglia from WT (black, *n* = 30) or V4KO (red, *n* = 30) mice. Data are represented as mean ± SEM. GSK indicates treatment with the TRPV4 activator GSK-1016790A (500 nM). Ionomycin (5 μM) was applied to assess cell viability. Bottom trace shows the heat stimulation. (*I*) Distances of migrating microglia isolated from WT or V4KO mice exposed to DMSO (1/1000) or GSK (500 nM) at 33 °C (WT, *n* = 156 for DMSO and *n* = 155 for GSK; V4KO, *n* = 151 for DMSO and *n* = 150 for GSK) or 37 °C (WT, *n* = 154 for DMSO and *n* = 151 for GSK; V4KO, *n* = 145 for DMSO and *n* = 150 for GSK). Filled circles indicate migration distance for each microglia cell over 1 h. Horizontal lines indicate mean ± SEM ***P < 0.001 (two-way ANOVA followed by post hoc Bonferroni comparison).

We next examined whether our candidate TRP channels are involved in temperature-dependent microglia movement in vitro using time-lapse imaging of microglia isolated from M2KO and V4KO mice. Both M2KO and V4KO microglia exhibited less movement than WT microglia at 37 °C and 40 °C, indicating that TRPM2 and TRPV4 could both be involved in temperature-dependent microglia movement (Fig. 3*A*). Notably, V4KO microglia, unlike M2KO microglia, did not exhibit temperature-dependent movement between 37 °C and 40 °C, suggesting different mechanisms for TRPM2- and TRPV4-induced microglia movement. This result suggested that thermosensitive TRPM2 and TRPV4 are involved in the mechanism of microglial motility in vitro, although TRPV4 could promote temperature-dependent microglia movement between 37 °C and 40 °C.

### Temperature Dependency of Microglia Movement Is Lost in the Presence of 9-Phenanthrol, which Equally Inhibits TRPM2, TRPV4, and TRPM4 Activity.

We also examined the involvement of TRPM4 activation in microglia movement using 9-phenanthrol, which is considered a specific TRPM4 inhibitor. Movement of microglia treated with 9-phenanthrol at 37 °C was dose-dependently inhibited with a half maximal inhibitory concentration (IC_50_) value of 23.4 μM, which is comparable to previously reported values (*SI Appendix*, Fig. S3 *A* and *B*) (26, 27). The temperature-dependency of WT microglia movement was also lost in the presence of 9-phenanthrol (30 μM) (Fig. 3*B*), suggesting that TRPM4 is involved in temperature-dependent microglia movement. Since 9-phenanthrol is reported to inhibit other non-TRP channels including transmembrane protein (TMEM)16A (28), we wondered about 9-phenanthrol specificity among the family of TRP channels and checked whether it could inhibit TRPM2 or TRPV4. Surprisingly, currents induced by GSK treatment were small in the presence of 9-phenanthrol and became larger upon 9-phenanthrol washout, although the magnitude of GSK-induced currents after the washout was typically smaller, possibly due to desensitization (Fig. 3 *C* and *D*). The IC_50_ values for inhibition of GSK-induced TRPV4 currents by 9-phenanthrol were 16.3 ± 5.3 μM (Fig. 3*E*), which is comparable to that previously reported for TRPM4 (26–28). In TRPV4-expressing human embryonic kidney-derived 293T (HEK293T) cells, heat-evoked currents were also smaller in the presence of 9-phenanthrol than those in its absence (Fig. 3*F*). In HEK293T cells expressing TRPM2, we also clearly observed dose-dependent inhibition of ADP ribose–induced currents by 9-phenanthrol (Fig. 3*G*) (*SI Appendix*, Fig. S3*C*). Taken together, 9-phenanthrol appears to inhibit not only TRPM4 but also TRPV4 and TRPM2 to the same extent, suggesting that 9-phenanthrol cannot be used to distinguish TRPV4, TRPM2, and TRPM4 channel activity. This possibility is consistent with the further inhibition of movement observed for TRPV4KO microglia treated with 9-phenanthrol at 37 °C and 40 °C (*SI Appendix*, Fig. S3*D*). Hence, 9-phenanthrol is not a specific inhibitor of TRPM4 channels, and it may act on TRPV4 and TRPM2 channels to inhibit temperature-dependent microglia movement. Then, we checked whether intracellular calcium was necessary for heat sensitivity of mouse microglia by a patch-clamp method. The density of heat-evoked currents observed in primary microglia was independent of intracellular free Ca^2+^ (1 μM; *SI Appendix*, Fig. S3*E*), suggesting that high levels of intracellular Ca^2+^ are not related to the mechanism for heat-evoked currents. As such, involvement of TRPM4 in microglia movement can be excluded.

To further discriminate between TRPV4 and TRPM2 channels, we also investigated the heat response of microglia in Ca^2+^ imaging experiments. WT microglia exhibited a clear intracellular Ca^2+^ increase upon heat stimulation, which was blunted in V4KO microglia (Fig. 3*H*), while M2KO microglia retained a strong response to both heat and GSK stimulations (*SI Appendix*, Fig. S3*F*). These results support the idea of a stronger contribution of TRPV4 to heat-induced responses in microglia. Next, we performed time-lapse imaging of microglia migration at 33 °C and 37 °C in the presence of GSK (Fig. 3*I*). In the presence of GSK, WT microglia exhibited enhanced migration at both temperatures compared with the dimethyl sulfoxide (DMSO) control, a phenomenon that was not observed in V4KO microglia. This result further supports our hypothesis of TRPV4 as the key ion channel for regulating microglia movement.

### TRPV4-Deficient Microglia Lose Temperature-Dependent Process Movement In Vivo.

**Fig. 4.**
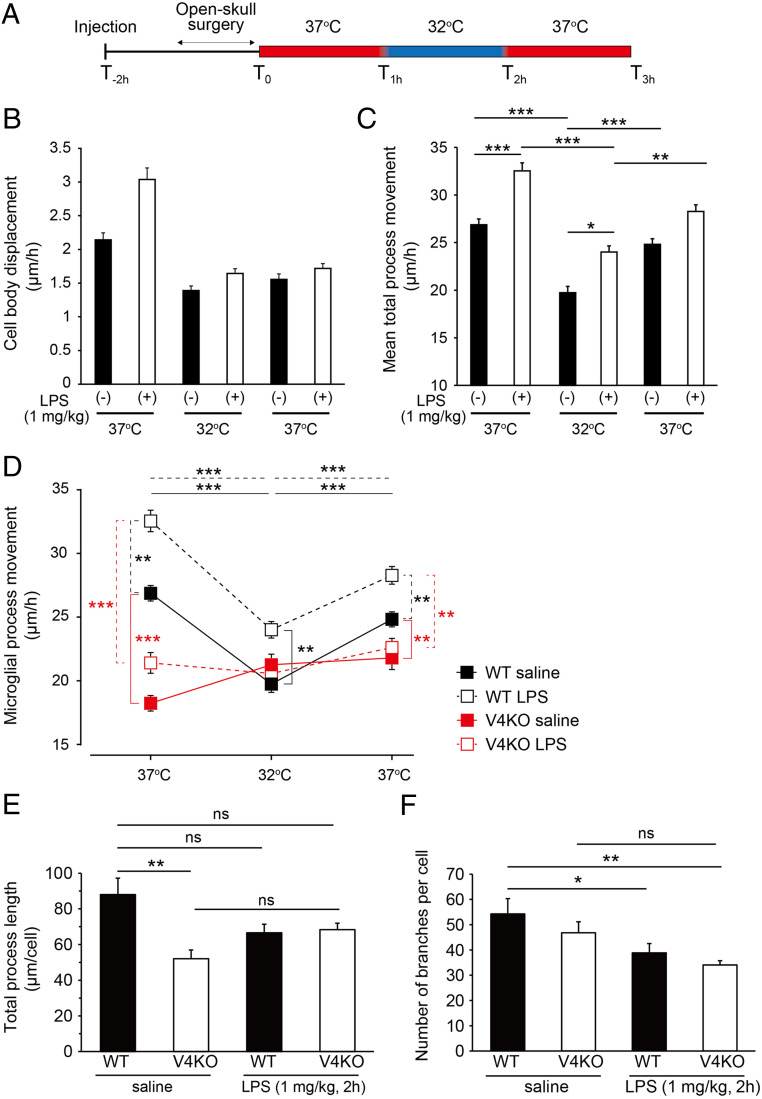
TRPV4-deficient microglia lose temperature-dependent process movement in vivo. (*A*) Experimental protocol for in vivo time-lapse imaging. Saline or LPS (1 mg/kg) was injected intraperitoneally 2 h prior to recordings. (*B*) Analysis of cell body displacement of WT microglia in the absence (saline injection) or presence of LPS. WT saline at 37 °C (first, *n* = 125 cells), 32 °C (*n* = 121), and 37 °C (second, *n* = 113) from three mice; WT LPS at 37 °C (first, *n* = 178 cells), 32 °C (*n* = 133), and 37 °C (second, *n* = 139) from three mice. Data are represented as mean ± SEM. ns, not significant (two-way ANOVA followed by post hoc Bonferroni tests for multiple comparisons). (*C*) Analysis of WT microglia process movement in the presence or absence (saline injection) of LPS. WT saline at 37 °C (first, *n* = 63 processes), 32 °C (*n* = 28), and 37 °C (second, *n* = 41) from three mice; WT LPS at 37 °C (first, *n* = 67 processes), 32 °C (*n* = 72), and 37 °C (second, *n* = 70) from three mice. Data are represented as mean ± SEM *P < 0.05, **P < 0.01, and ***P < 0.001 (two-way ANOVA followed by post hoc Bonferroni tests for multiple comparisons). (*D*) Comparison of movement of microglia processes from WT or V4KO mice in the absence (saline injection) or presence of LPS. WT saline at 37 °C (first, *n* = 63 processes), 32 °C (*n* = 28), and 37 °C (second, *n* = 41) from three mice; WT LPS at 37 °C (first, *n* = 67 processes), 32 °C (*n* = 72), and 37 °C (second, *n* = 70) from three mice; V4KO saline at 37 °C (first, *n* = 46 processes), 32 °C (*n* = 46), and 37 °C (second, *n* = 46) from three mice; V4KO LPS at 37 °C (first, *n* = 79 processes), 32 °C (*n* = 75), and 37 °C (second, *n* = 59) from three mice. Data are represented as mean ± SEM **P < 0.01, ***P < 0.001 (two-way ANOVA followed by post hoc Bonferroni tests for multiple comparisons). (*E*) Comparison of total process length of microglia from WT or V4KO mice in the absence (saline) or presence of LPS at 37 °C. WT saline (*n* = 52 cells) from four mice; WT LPS (*n* = 64 cells) from five mice; V4KO saline (*n* = 35 cells) from three mice; and V4KO LPS (*n* = 109 cells) from seven mice. Data are represented as mean ± SEM, **P < 0.01 (two-way ANOVA followed by post hoc Bonferroni tests for multiple comparisons). (*F*) Comparison of the number of branches per microglia cell from WT or V4KO mice in the absence (saline) or presence of LPS at 37 °C. WT saline (*n* = 52 cells) from four mice; WT LPS (*n* = 64 cells) from five mice; V4KO saline (*n* = 35 cells) from three mice; and V4KO LPS (*n* = 109 cells) from seven mice. Data are represented as mean ± SEM, *P < 0.05, **P < 0.01 (two-way ANOVA followed by post hoc Bonferroni tests for multiple comparisons).

TRPV4 appears to have greater importance than other TRP channels given that the distance moved by V4KO microglia was similar at 37 °C and 40 °C, and that WT microglia moved more in the presence of a TRPV4 agonist (Fig. 3 *A* and *I*). We next investigated whether microglia can detect temperature changes in vivo using two-photon microscopy in the absence (saline) or presence of lipopolysaccharide (LPS, 1mg/kg) injected 2 h prior to imaging, which served as a model of systemic inflammation to mimic microglial function in the pathology (29). Since microglia in vitro are thought to be activated upon isolation, we examined both temperature conditions in the absence and presence of LPS using the experimental protocol shown in Fig. 4*A*. After the open-skull surgery, the mouse was kept under anesthesia and then set on the stage for transcranial imaging 1 h after surgery. The rectal temperature was used as the core temperature and was monitored and maintained using a heating pad under the mouse while the brain temperature was controlled by a water perfusion system on the brain’s surface. As previously reported (3), there was little displacement of the microglia cell body itself following either saline or LPS injection (Fig. 4*B*). On the other hand, movement of microglial processes did show temperature-dependent changes (Fig. 4*C*), suggesting that this movement is temperature dependent in vivo. Moreover, the process movement was larger following LPS injection than that seen for saline injection. The process movement was not affected by application of tetrodotoxin (*SI Appendix*, Fig. S4*A*), indicating that, under our experimental conditions, these movements are not related to neuronal activity, which is consistent with a previous report (1). The temperature-dependent process movement was maintained in M2KO microglia and appeared to be more intense than for WT microglia (*SI Appendix*, Fig. S4*B*). In contrast, microglia temperature-dependent process movement was abolished upon Trpv4 knockout both in the presence and absence of LPS injection (Fig. 4*D*). We could not conduct in vivo imaging at higher temperatures (>39 °C), which were cytotoxic.

We further investigated the importance of the TRPV4 channel by assessing the change in microglia morphology between resting and activated states in WT and V4KO mice in vivo (Fig. 4 *E* and *F*). The total process length of V4KO microglia was significantly shorter compared with that of WT cells under control conditions (saline), during which microglia should be in the resting state. Moreover, WT microglia showed a tendency toward a shorter process length after an LPS injection, which is consistent with previous reports showing shortened processes after activation of microglia (30, 31). On the other hand, V4KO microglia did not exhibit a difference in process length after LPS stimulation. Similarly, the number of branches was significantly lower in WT microglia in an LPS condition compared with a saline condition, but this was not the case for V4KO microglia. These results could be explained by V4KO microglia lacking the Ca^2+^-dependent actin remodeling necessary for process extension and retraction and for the change in global morphology that are required for the temperature-dependent microglia movement. Collectively, our results suggest that TRPV4 activation is required for temperature-dependent microglia movement in vitro and in vivo. It should be noted that there was a difference in the temperature range at which V4KO microglia exhibited characteristic behaviors in vitro and in vivo, which should be further investigated.

## Discussion

Microglia are the resident immune cells in the CNS and are involved in virtually all pathological processes in the CNS, from infection and neurodegenerative diseases to mental disorders (2). In CNS disorders, microglia can have either detrimental or beneficial actions depending on extracellular context and thus have attracted substantial interest (32). Even though microglia are sensitive to their environment, how fluctuations in brain temperature can affect microglial functions has not been thoroughly investigated. Here we showed that microglia movement exhibits temperature-dependency, both in vitro and in vivo (Figs. 1 and 4). Displacement of microglia cell bodies was markedly affected by temperature in vitro, but in vivo temperature-dependent effects were largely restricted to microglial process movement. This result is not surprising since the amoeboid phenotype of microglia that exhibits the property of cell body displacement is rarely observed in vivo (1, 33). Nevertheless, it is possible that the lack of cell body displacement in vivo is linked to limitations in current observation techniques. Wake et al. previously reported that at 32 °C, the frequency of contacts between microglial processes and neuronal synapses was significantly reduced due to decreased neuronal activity rather than a decrease in the speed of microglial process movement (3). This result is in apparent contrast with our observation that the temperature-dependency of microglial process movement does not depend on neuronal activity (*SI Appendix*, Fig. S4*A*) but could be explained by differences in experimental models.

The range of temperatures used in our study mimics hypothermia (<33 °C) as well as physiological (37 °C) and pathological (40 °C) conditions. Brain temperature depends mainly on local heat production, temperature of the blood vessels, and cerebral blood flow, which can all vary to contribute to thermal changes (34). Physiological and pathological fluctuations in brain temperature can be as large as 3 °C or 4 °C (35), and therefore, it is likely that the temperature-dependent movement of microglia supports or perturbs the brain homeostasis under such circumstances. In particular, this temperature-dependent movement might have an important role during fever or brain injury when local brain temperatures are increased (36). During brain injury or infection, microglia are rapidly activated as part of an inflammatory response that is often accompanied by fever. It is already known that fever can enhance some responses in immune cells—for example, macrophages present higher bacterial clearance and cytokine production during whole-body heating (14, 37–39). Microglia, whose functions rely on their process motility and cell body movement, may in a similar manner present enhanced immune responses during fever. Temperature-dependent movement of microglia might be important for their optimal response to adverse events.

We observed that V4KO microglia lost their temperature-dependency under both physiological and pathological conditions in vivo (Fig. 4), suggesting that TRPV4 channels are a critical component of temperature-dependent microglia movement. At physiological brain temperature, TRPV4 is constitutively active and therefore could be an important source of Ca^2+^ that is required for proper microglia movement. TRPV4 channels are highly Ca^2+^ permeable and thus could provide a sufficient amount of Ca^2+^ to trigger actin cytoskeleton rearrangement and modulate focal adhesions (40, 41). Indeed, recent reports show that TRPV4 interacts with cytoskeletal elements, including actin, tubulin, and neurofilaments and that TRPV4 activity promotes actin polymerization through Rho GTPase activation (41–43). TRPV4-mediated Ca^2+^ influx also activates protein kinases and calpains involved in focal adhesion disassembly processes (44). Thus, TRPV4 plays an important role in cell migration. In pathological high-temperature ranges, TRPV4 channels could become overactive and in turn promote Ca^2+^-dependent cytoskeletal rearrangement and enhanced microglia movement. Whether this temperature-dependent enhancement in microglia movement is detrimental or beneficial for brain pathophysiology remains unclear but could depend on the pathological environment. For example, injured or inflamed regions of the brain could produce a local increase in temperature to attract more microglia via thermotaxis or facilitate their movement along a chemoattractant gradient formed by purine nucleotides released from the affected areas (45–47). Interestingly, neuronal excitability was also shown to be modulated by brain temperature in a TRPV4-dependent manner (15). TRPV4 activity at physiological temperatures is essential to maintain the resting membrane potential of hippocampal neurons close to the threshold for action potential firing. Together with this previous study, our observation that TRPV4 is required for proper microglia movement at physiological temperatures suggests that TRPV4 channels constitute an important molecular mediator of temperature fluctuations surrounding neurons and glial cells in the brain.

As described above, TRPV4 channels are expressed in several cell types including neurons and astrocytes in some parts of the brain, and body temperature can modulate the physiological function of such TRPV4-expressing cells (15, 21, 48). There are reports showing that microglia movement can be guided by an ATP gradient established by other cell types such as astrocytes (29, 45). It might suggest that cells other than microglia could be involved in the microglia movement phenotypes observed in our study possibly through the interaction of different cell types. Although we performed our study using conventional V4KO mice, cell type–specific knockout of Trpv4 would provide the supportive information especially regarding its relevance for organismal physiology and pathobiology in the near future.

Our in vitro time-lapse imaging experiments indicated a possible involvement of TRPM2, even though M2KO microglia in vivo still exhibited temperature-dependent process movement. These results suggest that, at least in our experimental model, TRPM2 could play a role in temperature-dependent microglia movement, although its role is less prominent than that of TRPV4. Given that TRPM2 is functionally expressed in microglia as we confirmed in this study, involvement of TRPM2 in microglia movement could also occur in other settings (49).

Results from the in vitro time-lapse imaging experiments using 9-phenanthrol as a supposedly specific TRPM4 inhibitor also suggested a potential role for TRPM4 channels in microglia movement (27). However, recent studies reported that 9-phenanthrol can inhibit other ion channels such as TMEM16A, a calcium-activated Cl^−^ channel (28). We found that 9-phenanthrol can inhibit TRPM2 and TRPV4 activity to the same extent as that seen for TRPM4 (Fig. 3 *C*–*G*). Moreover, the involvement of TRPM4 channels—which are activated by Ca^2+^ but are not Ca^2+^ permeable—in the temperature-dependent movement of microglia seems unlikely given that heat-activated currents were not affected by changes in intracellular Ca^2+^ concentrations (*SI Appendix*, Fig. S3*E*). Thus, we believe the contribution of TRPM4 to temperature-dependent movement of microglia is rather weak as this channel would require the cooperation of Ca^2+^-permeable channels. Although TRPM4 was shown to regulate Ca^2+^ influx by modulating the membrane potential, and thus maintain proper intracellular Ca^2+^ levels to allow migration in mast cells (50, 51), this mechanism relies on the activity of other channels responsible for Ca^2+^ entry. Furthermore, another study showed that depending on the level of expression of TRPM4 channels, it can differentially impact (i.e., promote or inhibit) cell motility (52). Therefore, TRPM4 contribution to temperature-dependent microglia movement is difficult to predict, and further investigation is needed.

Overall, the results of our study indicate that thermosensitive TRP channels, and particularly TRPV4, are strong modulators of microglial functions in response to brain temperature fluctuations. By understanding the mechanisms of microglia movement and how these mechanisms are modulated by temperature, we can identify leads for the treatment of CNS pathologies.

## Methods

### Animals.

C57BL/6NCr mice (SLC Japan) were used as the WT strain. M2KO, V4KO, and Iba1-EGFP mice were generously provided by Dr. Yasuo Mori (Kyoto University, Japan) (53), Dr. Makoto Suzuki (Jichi Medical School, Japan) (54) and Dr. Junichi Nabekura (National Institutes of Natural Sciences, Japan) (55), respectively. For in vitro time-lapse imaging, postnatal pups (P0 through P3) were obtained from each genotype. For in vivo two-photon imaging, male and female M2KO- or V4KO-Iba1-enhanced green fluorescent protein (EGFP) mice (8- to 14-wk-old) were obtained from a mating between each type of knockout mouse and Iba1-EGFP mice. Mice were housed in a controlled environment (12 h light/12 h dark cycle; room temperature, 22 °C to 24 °C; 50 to 60% relative humidity) with free access to food and water. All procedures involving the care and use of animals were approved by the Institutional Animal Care and Use Committee of the National Institutes of Natural Sciences and carried out in accordance with the NIH Guide for the care and use of laboratory animals (NIH publication no. 85–23, revised 1985).

### Preparation of Primary Mouse Microglia.

Primary mouse microglia were obtained according to a previously described method (46, 56) with modification. Mixed glial cultures were prepared from cerebral hemispheres of postnatal (P0 through P3) pups. The tissues were minced and trypsinized with DNase (Roche) at 37 °C for 10 min before horse serum (Biowest) was added. Dissociated cells were seeded as mixed glia cultures in 75 cm^2^ tissue flasks in D-MEM (Dulbecco's Modified Eagle Medium) (Sigma-Aldrich), with 10% (volume/volume) heat-inactivated bovine serum (Sigma-Aldrich), penicillin–streptomycin (10 units/mL and 10 mg/mL, respectively, Gibco), bovine insulin (5 μg/mL, Sigma-Aldrich), and glucose solution (2 mg/mL, Otsuka) and cultured for 10 to 20 d at 37 °C in a humidified CO_2_ incubator until the cells were 100% confluent. Primary microglia were obtained by shaking the flasks containing the mixed glia cultures and the supernatant with floating cells. Cells were seeded on 35 mm glass bottom dishes or 12 mm cover glasses (Matsunami Glass) at a density of 1 × 10^4^ cells/dish and incubated at 37 °C in a humidified CO_2_ incubator. Cells were used for experiments within 6 d of isolation.

### Expression Vectors.

Mouse TRPM2 cDNA in the pCIneo expression vector, mouse TRPV4 complementary deoxyribonucleic acid (cDNA) in the pcDNA3 expression vector, and mouse TRPM4 cDNA in the pcDNA3 expression vector were generously provided by Dr. Yasuo Mori (Kyoto University, Japan), Dr. Michael Caterina (Johns Hopkins University), and Dr. Veit Flockerzi (Universität des Saarlandes, Germany), respectively. The entire sequence of these vectors was confirmed by DNA sequencing.

### Cell Culture and Transfection.

HEK293T cells were maintained in D-MEM (Wako) supplemented with 10% (volume/volume) fetal bovine serum (BioWest or Gibco), penicillin–streptomycin (50 units/mL and 50 μg/mL, respectively, Gibco) and GlutaMAX (2 mM, Gibco) and seeded at densities of 5 × 10^5^ cells per 35 mm dish 24 h before transfection. For patch-clamp recordings, HEK293T cells cultured in OPTI-MEM I medium (Invitrogen) on 35 mm dishes were transfected with 1 μg expression vector and 0.1 μg pGreen-Lantern 1 cDNA (pGL) using Lipofectamine (Invitrogen) and Plus reagents (Invitrogen) according to the manufacturer’s protocol. After incubation for 3 to 5 h, cells were reseeded on cover glasses and further incubated at 37 °C in a humidified 5% CO_2_ incubator. Cells were used for experiments 20 to 36 h after transfection.

### In Vitro Time-Lapse Imaging of Microglia Movement.

Glass bottom dishes containing primary mouse microglia were placed on the stage of an inverted Keyence BZ-9000 (Keyence) fluorescence microscope fitted with a temperature-controlled stage incubator (Tokai Hit) under a circulating mixture of gases (20% O_2_, 5% CO_2_, and 75% N_2_). The stage/cover temperatures were set at 33/33 °C (33 °C), 37/40 °C (37 °C), or 40/41 °C (40 °C) for each experiment at least 1 h before cell image acquisition except for the experiment involving sequential temperature changes (Movie S1). The temperature setting for each condition was determined in advance by measuring the medium temperature with a wire probe and a digital thermometer (Unique Medical). Cells were imaged at 1 or 3 min intervals with a 20× phase contrast objective lens (Nikon Instech). Image acquisition was performed using BZ-9000 software with a Z-stack function. Focused images taken with the Z-stack function were automatically selected using a BZ-II analyzer (Keyence) in each frame correction and then reconstructed into a series of stacking images along the time axis. In experiments that included 9-phenanthrol, images were captured for 2 h for the control, and then 9-phenanthrol at the indicated concentration was added to the medium at a dilution of 1:1,000. The migration distances of microglia were analyzed using ImageJ (NIH) and the Manual Tracking and Chemotaxis and Migration plugin tool. Excel software (Microsoft) was used to calculate the migration distances of individual cells. Data defining the migration distances of microglia were collected from at least three individual glass bottom dishes and three different preparations by analyzing all cells or randomly analyzing ∼50 cells per dish (Fig. 3*I*).

### RT-PCR.

Total RNA was purified from primary mouse microglia using an RNeasy Plus Mini kit (Qiagen) with DNase I (Sigma-Aldrich) treatment to eliminate genomic DNA according to the manufacturer’s protocols. cDNA was synthesized from total RNAs (up to 1 μg) using reverse transcription with a SuperScript III first-strand synthesis system for RT-PCR (Invitrogen). PCR was performed using rTaq polymerase (TaKaRa) with an iCycler instrument (Bio-Rad) and specific primer sets. Forward and reverse primers were 5′-TGA​AGG​GTG​GAG​CCA​AAA​GG-3′ and 5′-GGA​AGA​GTG​GGA​GTT​GCT​GTT​G-3′ for Gapdh (57), 5′-CGG​CTT​CAG​AGA​TGA​CCA​G-3′ and 5′-GCT​TCA​TTC​ATC​ATG​TCC​TTG-3′ for Cd11b, 5′-AAC​TCC​ACC​CCA​CAC​TGA​AG-3′ and 5′-TCG​CCT​CTG​CAG​GAA​ATA​CT-3′ for Trpv1 (57), 5′-ACC​GCA​TGG​TGG​TTT​TAG​AG-3′ and 5′-CTA​CAG​CAA​AGC​CGA​AAA​GG-3′ for Trpv2 (57), 5′-TGA​CAG​AGA​CCC​CAT​CCA​AT-3′ and 5′-GGG​TGT​CTC​GCC​AAA​ATA​GA-3′ for Trpv3, 5′-ACA​ACA​CCC​GAG​AGA​ACA​CC-3′ and 5′-CCC​AAA​CTT​ACG​CCA​CTT​GT-3′ for Trpv4 (57), 5′-ACA​ACC​CTG​AAG​GAC​AGT​GG-3′ and 5′-CAT​CAC​TAG​CAC​CTC​CAG​CA-3′ for Trpm2 (57), 5′-TGA​CCA​AGG​AAT​GGC​AAC​TG-3′ and 5′-ATG​CCA​GGA​TGT​CGG​ATG-3′ for Trpm3, 5′-TGG​ATG​CTC​TGC​TGA​ATG​AC-3′ and 5′-GAC​TCT​AGG​CGA​GCC​ATC​AC-3′ for Trpm4 (57), and 5′-CTG​ATC​GCC​ATG​TTC​AGC​TA-3′ and 5′-GGA​GCC​AGT​GTA​TCC​GTC​AT-3′ for Trpm5 (57). PCR conditions were the following: one cycle at 94 °C for 2 min; 35 cycles at 94 °C for 10 s, 58 °C for 10 s, and 72 °C for 30 s; and one cycle at 72 °C for 2 min. The specificity of the amplified fragments was confirmed by sequencing. Confirmed fragments were used as a positive control.

### Protein Expression Assay.

Samples were prepared from HEK293T cells expressing mouse TRPV4 or naïve HEK293T cells for the control. Samples were also prepared from primary mouse microglia isolated from WT mice. HEK293T cells or primary mouse microglia were homogenized in radioimmunoprecipitation assay (RIPA) lysis buffer with protease inhibitor mixture (Cell Signaling Technology) and lysed. For Western blot experiments, 20 to 40 µg protein was used. The proteins were denatured with sodium dodecyl sulfate (SDS) buffer containing 50 mM Tris-HCl, 2% SDS, 100 mM dithiothreitol (DTT), 10% glycerol, and 0.01% bromophenol blue at 95 °C for 5 min before resolution by SDS-polyacrylamide gel electrophoresis (PAGE). After transfer to a polyvinylidene difluoride (PVDF) membrane (Merck Millipore, IPVH00010), the membrane was blocked for 1 h at room temperature and then incubated with anti-TRPV4 antibody (abcam, ab39260) (1:1,000), anti-TRPM2 antibody, a gift from Y. Mori (Kyoto University, Kyoto, Japan) (1:200), or anti-TRPM4 antibody, a gift from V. Flockerzi (Universitat des Saarlandes, Saarbrücken, Germany) (1:200) overnight at 4 °C. After three washes with phosphate buffered saline with Tween 20 (PBS-T), the membrane was incubated for 1 h at room temperature with either an anti-rabbit or an anti-mouse IgG horseradish peroxidase (HRP)-linked antibody (1:1,000, Cell Signaling Technology). After incubating the membrane for 5 min with enhanced chemiluminescence reagent (GE Healthcare, RPN2232), proteins were detected with an imager (Fujifilm, LAS-3000). The digital images were then quantified using Adobe Photoshop and ImageJ.

### Ca^2+^ Imaging.

Primary mouse microglia from WT, M2KO, or V4KO mice on coverslips were incubated at 37 °C for 30 min in culture medium containing 5 μM fura-2-acetoxymethyl ester (Molecular Probes). The coverslips were washed with a standard bath solution that was identical to the standard solution used in the patch-clamp recordings as described below. Fura-2 fluorescence was measured in a standard bath solution with Fura-2 excitation wavelengths of 340 and 380 nm and monitoring of fluorescence emission at 510 nm with a CoolSnap ES charge coupled device (CCD) camera (Roper Scientific/Photometrics) at room temperature. GSK-1016790A (GSK) was applied as described below for the patch-clamp recordings. Data were acquired using IPlab software (Scanalytics) and analyzed with ImageJ (NIH) and Excel software (Microsoft). To prepare the Ca^2+^ (−) extracellular solution, 5 mM EGTA was added to the standard bath solution without Ca^2+^. Ionomycin (5 μM, Sigma-Aldrich) was applied to confirm cell viability.

### Electrophysiology.

Transfected HEK293T cells or primary mouse microglia on cover glasses were incubated in culture medium at 37 °C. For experiments shown in Fig. 3*F*, cells were incubated at 33 °C for 24 h prior to beginning the experiment to induce overactivation. The cover glasses were mounted in a chamber (Warner Instruments) connected to a gravity flow system to deliver various stimuli and heated bath solutions. The cover glasses were washed with a standard bath solution containing 140 mM NaCl, 5 mM KCl, 1 mM MgCl_2_, 2 mM CaCl_2_, 10 mM Hepes, and 10 mM glucose at pH 7.4 adjusted with NaOH. For experiments shown in Fig. 2 *E* and *F* and *SI Appendix*, Figs. S2 and S3*E*, the extracellular solution was replaced after obtaining a whole-cell configuration in a K^+^-free bath solution containing 145 mM NaCl, 1 mM MgCl_2_, 2 mM CaCl_2_, 10 mM Hepes, and 10 mM glucose at pH 7.4 adjusted with NaOH. The intracellular solution for whole-cell recordings contained 20 mM NaCl, 120 mM Na gluconate, 1 mM MgCl_2_, 5 mM EGTA, and 10 mM Hepes at pH 7.3 adjusted with NaOH. For experiments in which the intracellular free calcium ([Ca^2+^]_i_) concentration was fixed at 1 μM, 4.53 mM CaCl_2_ was added (calculated by Maxchelator, standard version) before the pH was adjusted. For experiments shown in Fig. 2 *C* and *D*, the extracellular solution contained 140 mM Na gluconate, 5 mM K gluconate, 2 mM Mg D-gluconate, 2 mM Ca gluconate, 10 mM Hepes, and 10 mM glucose at pH 7.4 adjusted with NaOH. The intracellular solution contained 140 mM K gluconate, 5 mM EGTA, and 10 mM Hepes at pH 7.4 adjusted with KOH. For experiments shown in Fig. 3 *C* and *G*, the extracellular solution was the same as the standard bath solution, and the intracellular solution contained 140 mM CsCl instead of K gluconate and was adjusted to pH 7.4 with CsOH. Data for analyses were sampled at 10 kHz and filtered at 5 kHz for whole-cell recordings (Axon 200B amplifier with pCLAMP software, Molecular Devices). Thermal stimulation was applied by increasing the bath temperature using a preheated solution delivered through an inline heater (Warner Instruments). The bath temperature in the chamber during recordings was monitored with a thermocouple (Warner Instruments) and sampled with an analog-to-digital converter with pCLAMP software (Molecular Devices). Membrane potential was clamped at −60 mV for TRPV4- and TRPM2-expressing HEK 293T cells, at 0 mV for TRPM4-expressing HEK293T cells (*SI Appendix*, Fig. S2), at −60mV for mouse microglia (Fig. 2 *C* and *D*), or −70 mV for mouse microglia shown in Fig. 2 *E* and *F* and *SI Appendix*, Fig. S2. Ramp pulses from −100 to 100 mV for 500 msec were applied every 5 s except for Fig. 2*D*, wherein ramp pulses were applied every 3 s. All experiments were performed at room temperature except for those involving thermal stimulation.

### In Vivo Two-Photon Imaging.

Iba1-EGFP, M2KO-, or V4KO-Iba1-EGFP mice (8 to 14 wk-old) were deeply anesthetized with ketamine (0.13 mg/g, i.p.) and xylazine (0.01 mg/g, intraperitoneal injection [i.p.]), and the head surface was cleaned of fur and skin to allow a custom-made, thin metal imaging frame to be secured to the skull using acrylic glue (Aron Alpha, Konishi) and dental acrylic cement (Quick Resin, SHOFU). The frame had a central hole that provided access to the skull and allowed the mice to be secured under the microscope during subsequent imaging. Under isoflurane anesthesia, a craniotomy was performed above the ipsilateral S1, and this small region (diameter < 2 to 3 mm) of the brain was covered with a glass coverslip (diameter 4.0 mm, thickness 0.12 mm, Matsunami Glass). Dental acrylic cement and adhesive glue were used to secure the coverslip to the surrounding skull and metal frame. A plastic chamber (diameter 3.5 cm) with a central hole having the same diameter as the imaging window was set atop the skull and fixed with dental acrylic cement and adhesive glue. A perfusion line connected to a water bath allowed the warming (37 °C) or cooling (32 °C) of the brain surface through perfusion of temperature-controlled water into the plastic chamber. During recordings, the core body temperature was monitored and maintained with a heating pad. The temperature setting for each condition was determined in advance by directly measuring the temperature of the cortical area with a digital thermometer (Chino) coupled to a sheathed thermocouple probe (Chino), while the rectal temperature was taken with a digital thermometer (Unique Medical). In Fig. 4*A*, 2 h prior to recordings, mice were injected with either saline or LPS (i.p, 1 mg/kg). Tetrodotoxin was applied to the cortical surface before application of the coverslip on the cranial window (*SI Appendix*, Fig. S4*A*). Images were acquired every 5 min over a 100 μm–thick window. The collected data were analyzed using Fiji (NIH). First, the Registration plugin was applied to correct three-dimensional drift over time. Then, the Trackmate plugin was used to track cell body movement in imaged ZT stacks. To analyze process movement, a two-dimensional max-intensity projection of a 25 μm imaged volume was produced for each stack, and one to two processes from each cell present in the field were manually tracked over time. To analyze the total process length and the number of branches, a two-dimensional max-intensity projection of a 25 μm imaged volume was produced for each stack, which was subjected to binary conversion followed by skeletonization, and analysis of the generated skeletons was then performed. Data were collected from at least three mice.

### Chemicals.

Stock solutions were prepared by dissolving 9-phenanthrol (9-phe, Tocris) and GSK 1016790A (GSK, Tocris) in DMSO. Cyclic ADP ribose was freshly dissolved on each experiment day. Tetrodotoxin (Wako) was dissolved in distilled water to prepare stock solutions. LPS were purchased from Sigma-Aldrich. The dissolved chemicals were diluted 1:1,000 in the medium and solution for time-lapse imaging and patch-clamp experiments, respectively. The final DMSO concentration did not exceed 0.1%.

### Statistical Analysis.

Data are presented as mean ± SEM. The abbreviation n indicates the number of data points. All statistical analyses were performed using Origin software (OriginLab). Student’s *t* test (*SI Appendix*, Fig. S3*A*), Welch’s *t* test (*SI Appendix*, Fig. S2*D*) or one-way (Fig. 1*C*) (*SI Appendix*, Fig. S3 *C* and *D*) or two-way (Figs. 3 *A*, *B*, and *I* and 4 *B–D*) (*SI Appendix*, Fig. S4 *A* and *B*) ANOVA with post hoc analysis Bonferroni test were applied as indicated for statistical analysis. *P* < 0.05 was considered significant. Data related to Fig. 3*E* and *SI Appendix*, Fig. S3*B* were fitted by a logistic curve to calculate the IC_50_ value.

## Supplementary Material

Supplementary File

Supplementary File

## Data Availability

All study data are included in the article and/or supporting information.
