# Supplementary material for: Thermosensitive TRPV4 channels mediate temperature-dependent microglia movement
Source: Proc Natl Acad Sci U S A. 2021 Apr 22;118(17):e2012894118. doi: 10.1073/pnas.2012894118 (PMC8092382; doi:10.1073/pnas.2012894118)

Supporting Information

Supporting figures, table and movie legends

Figure S1. Protein expression of candidate TRP channels and TRPV4-dependent intracellular Ca^{2+} increases in mouse microglia

(A, B, C) Western blots for three TRP channels. Expected molecular weights for **(A)** TRPV4, **(B)** TRPM2 and **(C)** TRPM4 are 98 kDa, 171 kDa and 134 kDa, respectively, in mouse microglia. **(D)** Mean calcium imaging traces in primary WT microglia (n = 30 cells). Microglia were incubated in a Ca^{2+}-free buffer (Ca^{2+} (-)) 30 sec after the start of the recording and then stimulated with a TRPV4 activator, GSK1016790A (GSK, 500 nM). Buffer was then replaced with one containing 2 mM Ca^{2+} (Ca^{2+} (+)) and GSK. Data are represented as mean ± SEM. Ionomycin (5 μM) was applied to assess cell viability.

Figure S2. Reversible inhibition of mouse TRPM4 (mTRPM4)-mediated heat responses by 9-phenanthrol.

(A,B) HEK293T cells expressing mouse TRPM4 (mTRPM4) dialyzed with intracellular-free Ca^{2+} solution (1 μM) were exposed to repeated heat stimuli with ramp-pulses (-100 to +100 mV in 500 msec, every 5 sec). The mTRPM4-mediated currents elicited in response to the second heat stimulation were recorded in the absence **(A)** or presence **(B)** of 9-phenanthrol (100 μM). **(C)** A representative whole-cell trace of heat-evoked currents in wild-type (WT) microglia in the presence of 9-phenanthrol (100 μM) with ramp pulses from -100 mV to +100 mV delivered every 5 sec. Lower trace indicates temperature transition. Vm = -70 mV. **(D)** Inhibition ratio by 9-phenanthrol (9-phe) (1,10 and 100 μM) of heat-evoked currents in WT microglia at -100 mV and +100 mV. The maximal currents before washout of 9-phenanthrol were divided by the initial currents before application of 9-phenanthrol, and the values were normalized to the values in the absence of 9-phenanthrol and shown as ratios. *P< 0.05, **P< 0.01 (Welch's *t*-test).

Figure S3. Dose-dependent inhibition of microglia movement and ADP ribose-induced mouse TRPM2 activation by 9-phenanthrol.

(A) Dose-dependent inhibition of WT microglia movement *in vitro* by 9-phenanthrol (9-phe).

Time-lapse images for each concentration of 9-phenanthrol (1 μM, n=97; 3 μM, n=124; 10 μM, n=155; 30 μM n=164; 100μM, n=87) were acquired over 2h at 37 °C. Data are represented as mean ± SEM. **, P < 0.01 (Student *t*-test). **(B)** The calculated IC_{50} value based on data in (A) was 23.4 μM. **(C)** Dose-dependent inhibition of ADP ribose-induced currents by 9-phenanthrol treatment of HEK293T cells expressing mouse TRPM2. ADP ribose (10 μM) was added to the pipette solution prior to obtaining the whole cell configuration. The percentage of inhibition at +100 mV was calculated by dividing the current immediately before washout of 9-phenanthrol and maximal currents immediately after washout (10 and 30 μM) or 30 seconds after washout (1 and 3 μM). n=6-12. Data are represented as mean ± SEM. ** P < 0.01, *** P < 0.001 (one-way ANOVA). **(D)** Average distances of migrating microglia isolated from WT or V4KO microglia exposed to 37 °C or 40 °C, in the presence or absence of 9-phenanthrol (30 μM), for 1h. WT at 37 °C (n = 53), V4KO at 37 °C (n = 58), V4KO + 9-phe at 37 °C (n= 20), WT at 40 °C (n = 47), V4KO at 40 °C (n = 70) and V4KO + 9-phe at 40 °C (n= 20). Data are represented as mean ± SEM. *, P < 0.05, **, P < 0.01 (one-way ANOVA followed by *post hoc* Bonferroni tests for multiple comparisons). **(E)** Comparison of heat-evoked current density in microglia in the presence (n = 10) or absence (n = 8) of high intracellular Ca^{2+} (1 μM). ns, not significant (Welch's t-test). **(F)** Calcium imaging trace in M2KO microglia. Data are represented as mean ± SEM. GSK indicates treatment with the TRPV4 activator GSK-1016790A (500 nM). Ionomycin (5 μM) was applied to assess cell viability. Bottom trace shows the heat stimulation.

Figure S4. Neuronal activity or TRPM2 activity is not required for temperature-dependent microglial process movement.

(A) *In vivo* time-lapse imaging of microglia movement was performed in the absence (black) or presence (red) of tetrodotoxin 10 μM using microglia from WT mice. Saline n = 48 from 3 mice; TTX, n = 25-33 from 2 mice. Data are represented as mean ± SEM. ns, not significant. **(B)** Temperature-dependent movement of microglial process was compared for microglia from *Trpm2*-knockout (blue, M2KO; n=34 from 4 mice) and WT (black; n=14 from 2 mice) mice. Data are represented as mean ± SEM. ns, not significant, *, P < 0.05 (two-way ANOVA).

Movie S1. *In vitro* time-lapse imaging of WT microglia exposed to sequential temperature changes in relation to Fig 1.

Time-lapse imaging of WT microglia was performed with sequential changes in temperature, which are shown in the upper right corner. The elapsed time (hh:mm:ss) is shown at the bottom

right corner. Scale bar, 50 μm.

Supplemental Figure 1

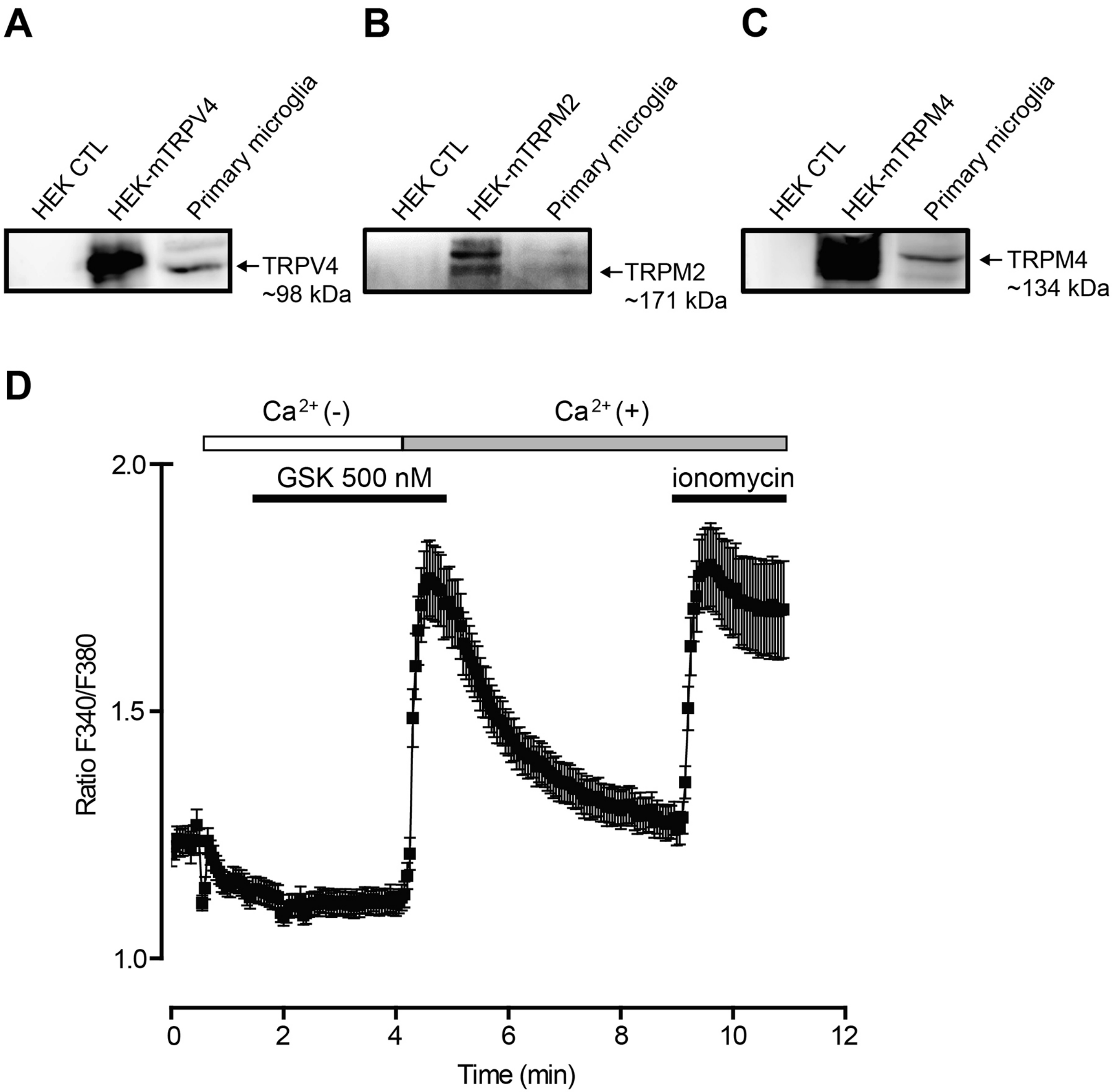

Supplemental Figure 2

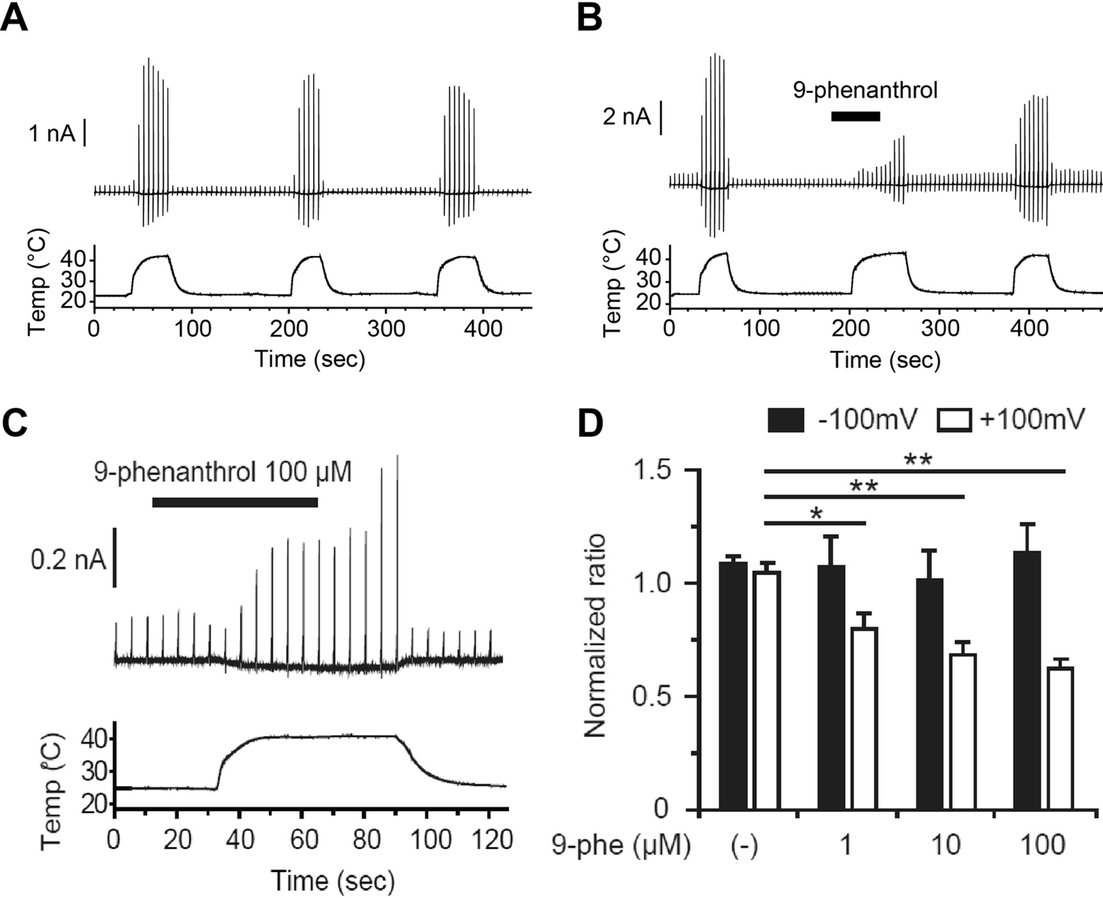

Supplemental Figure 3

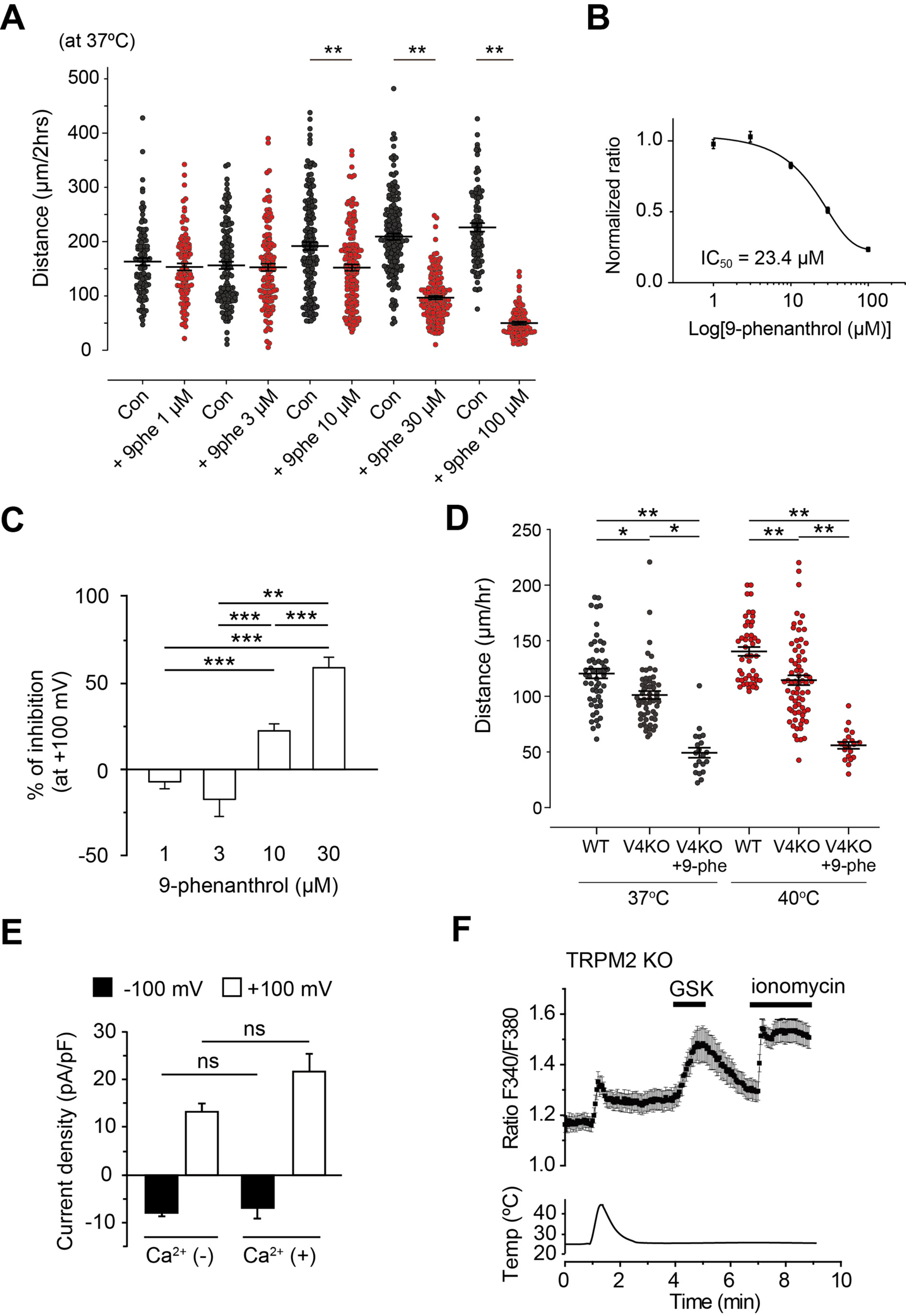

Supplemental Figure 4

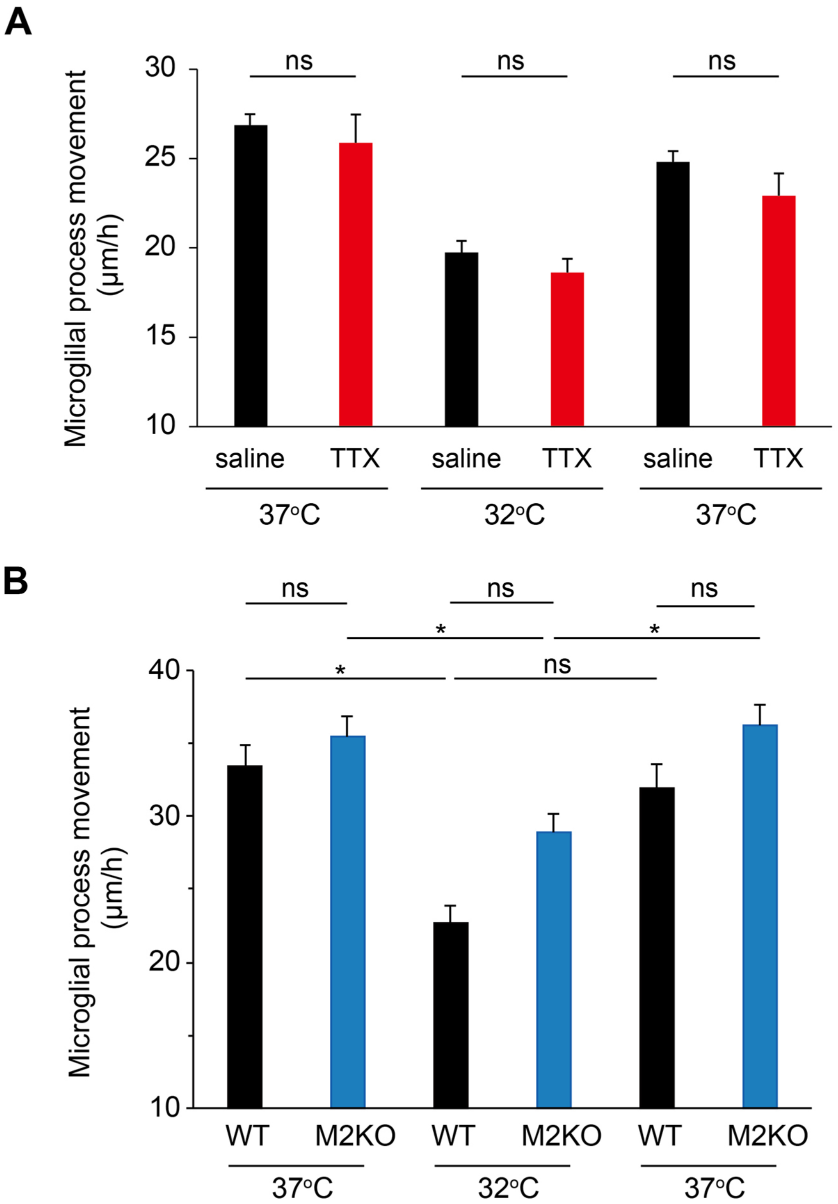

Supplement: Supplementary File [file pnas.2012894118.sapp.pdf]
